# Supplementary material for: Comprehensive single-cell atlas of the mouse retina
Source: iScience. 2024 May 8;27(6):109916. doi: 10.1016/j.isci.2024.109916 (PMC11134544; doi:10.1016/j.isci.2024.109916)
Supplement: Document S1. Figures S1‒S11 [file mmc1.pdf]

iScience, Volume *27*

Supplemental information

Comprehensive single-cell

atlas of the mouse retina

Jin Li, Jongsu Choi, Xuesen Cheng, Justin Ma, Shahil Pema, Joshua R. Sanes, Graeme Mardon, Benjamin J. Frankfort, Nicholas M. Tran, Yumei Li, and Rui Chen

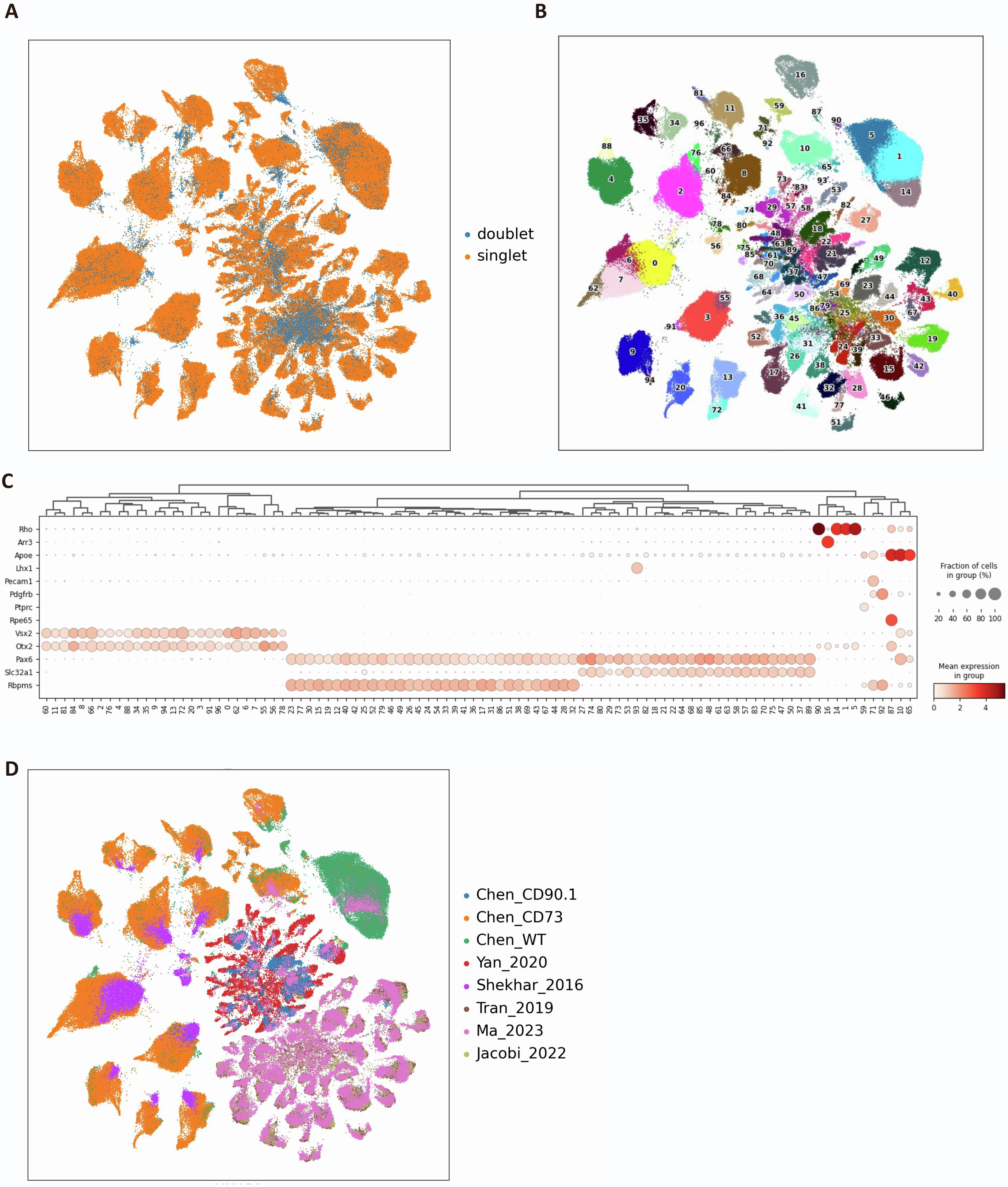

Figure S1. Cell type annotation of major classes. Related to Figure 1.

(A) UMAP plot of the integrated data colored by doublet status using SOLO algorithm. Doublets are labeled in blue, while singlets are labeled in orange. **(B)** UMAP plot of the integrated data colored by each Leiden cluster. The Leiden cluster ID is placed on top of each cell cluster. **(C)** Dot plot of expression patterns of canonical markers of major classes in each Leiden cluster. Marker gene expression allows the annotation of 11 major classes. **(D)** UMAP plot of the integrated data colored by data sources. An even distribution of cells across clusters is shown by the expected data source.

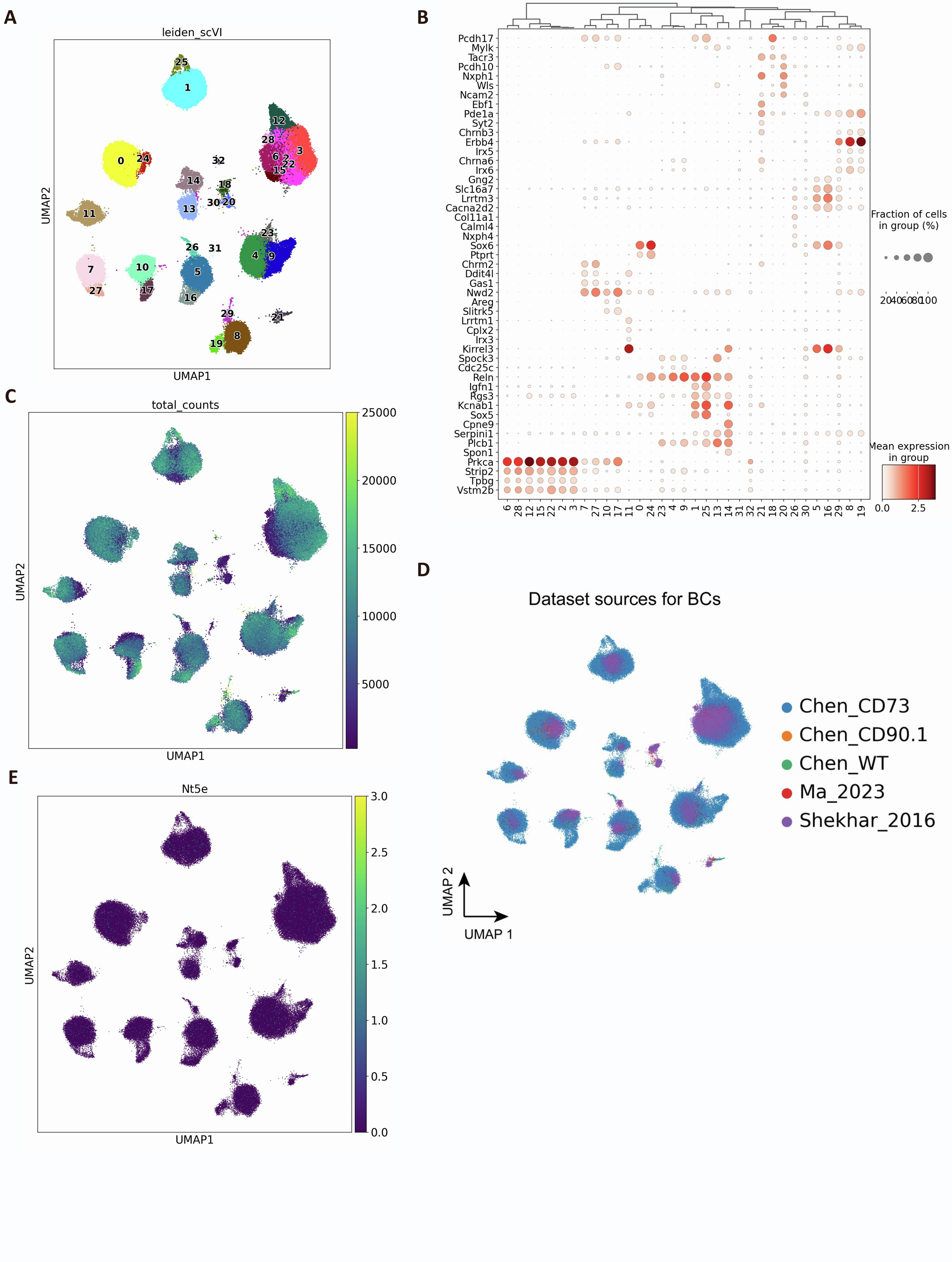

Figure S2. Cell type annotation of bipolar cells. Related to Figure 2.

(A) UMAP visualization of BCs colored by cell clusters. **(B)** Dot plot of BC type marker gene expression in each cluster. **(C)** UMAP plot of BCs colored by the total UMI counts. **(D)** UMAP plot of BCs colored by data sources. **(E)** UMAP plot of *Nt5e* (CD73) expression in BCs.

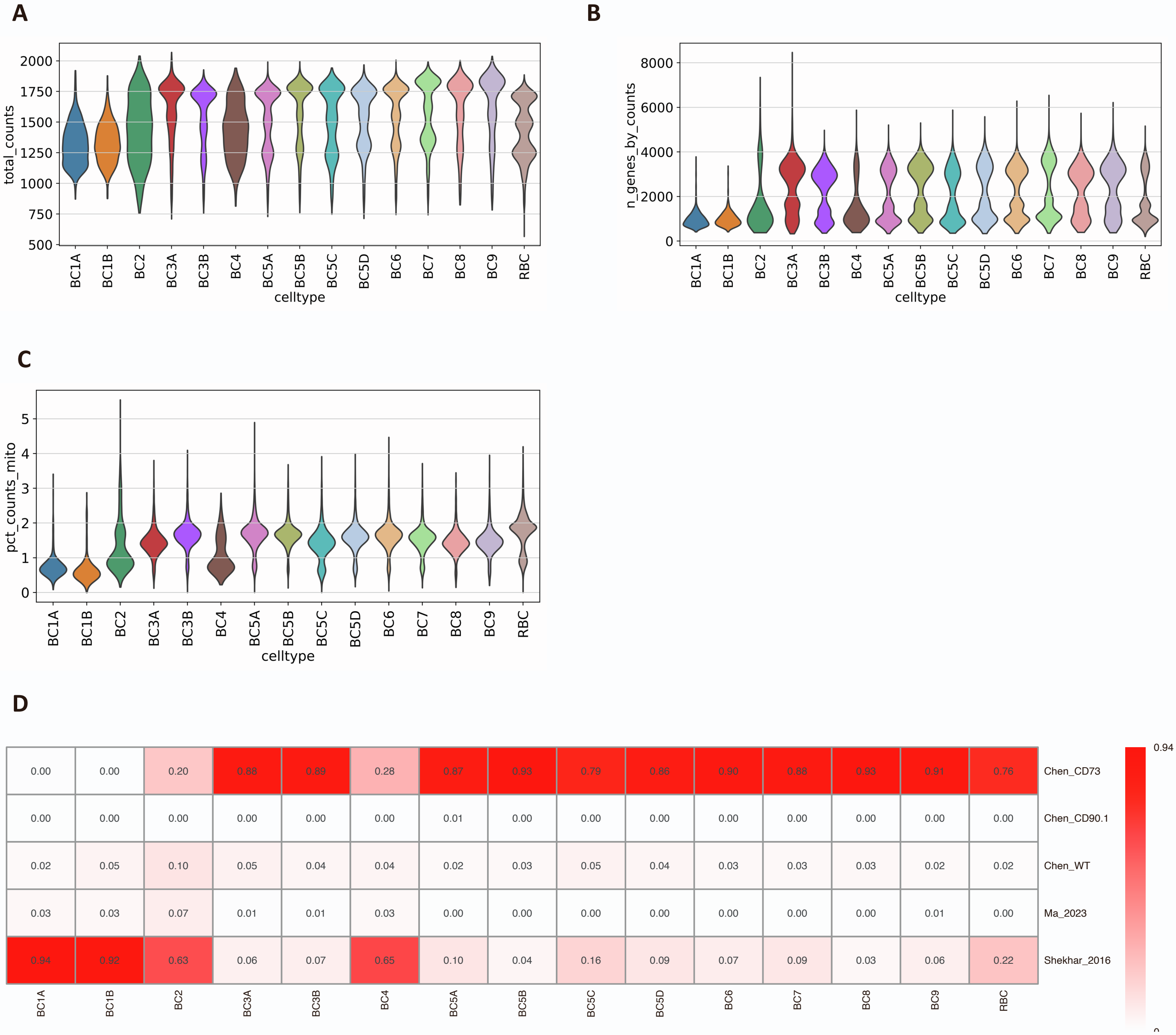

Figure S3. Quality control of BC types. Related to Figure 2.
Violin plots display the distribution of total UMI counts (**A**), the number of features (**B**), and the percentage of mitochondrial counts (**C**) for the annotated BC types. (**D**) The percentage of dataset sources for BC types. Each row represents a dataset source, while each column represents a BC type. The numbers in the table indicate the percentage of the dataset source for each cell type. The color key denotes the percentages.

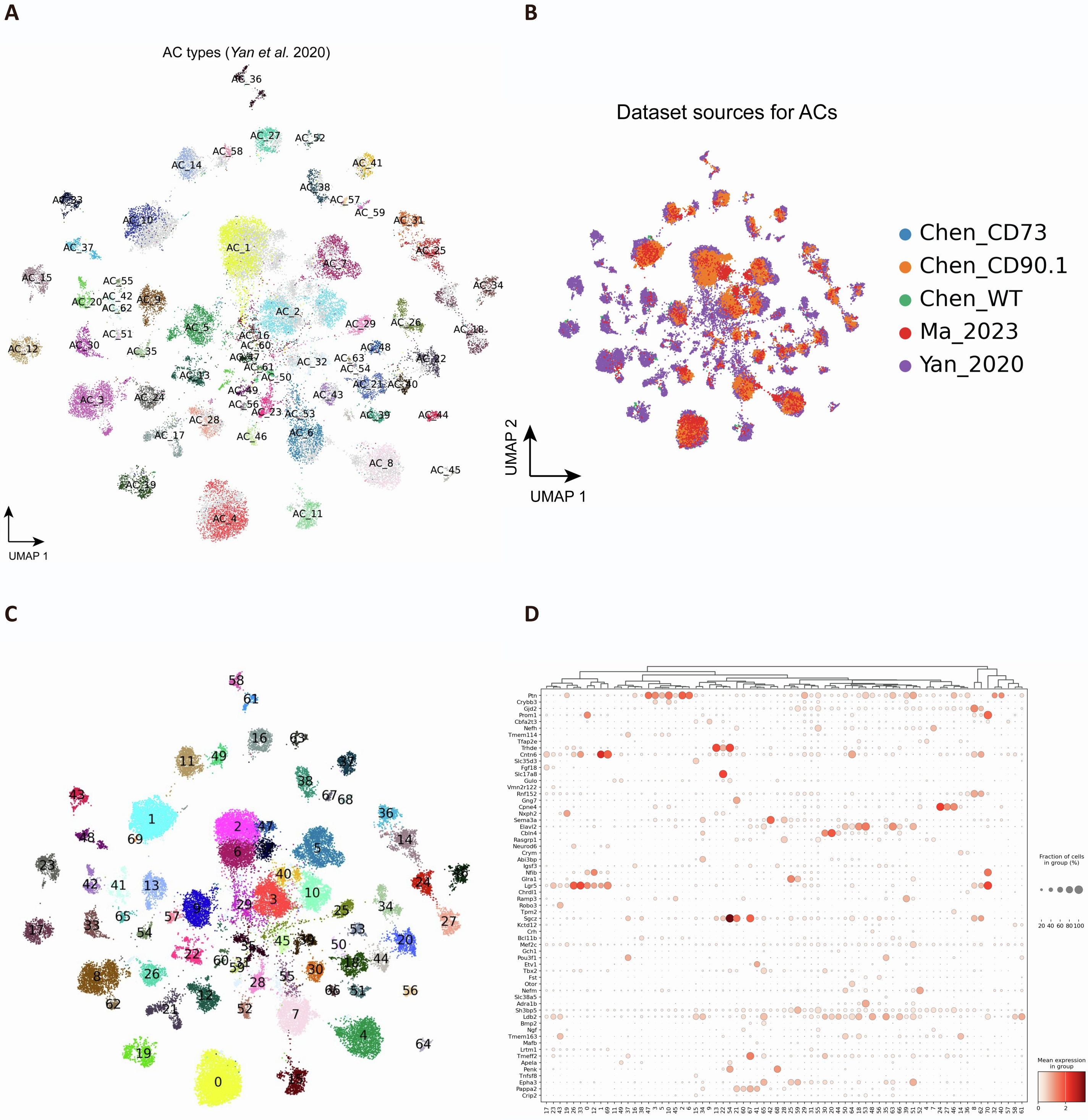

Figure S4. Cell type annotation of amacrine cells. Related to Figure 3 and Figure 4.
(A) UMAP visualization of ACs colored by public cell type labels from *Yan et al.* 2020. The newly discovered cells without public labeling are colored in gray. **(B)** UMAP plot of ACs colored by data sources. **(C)** UMAP visualization of ACs colored by 71 cell clusters. **(D)** Dot plot of AC type marker gene expression in 71 clusters.

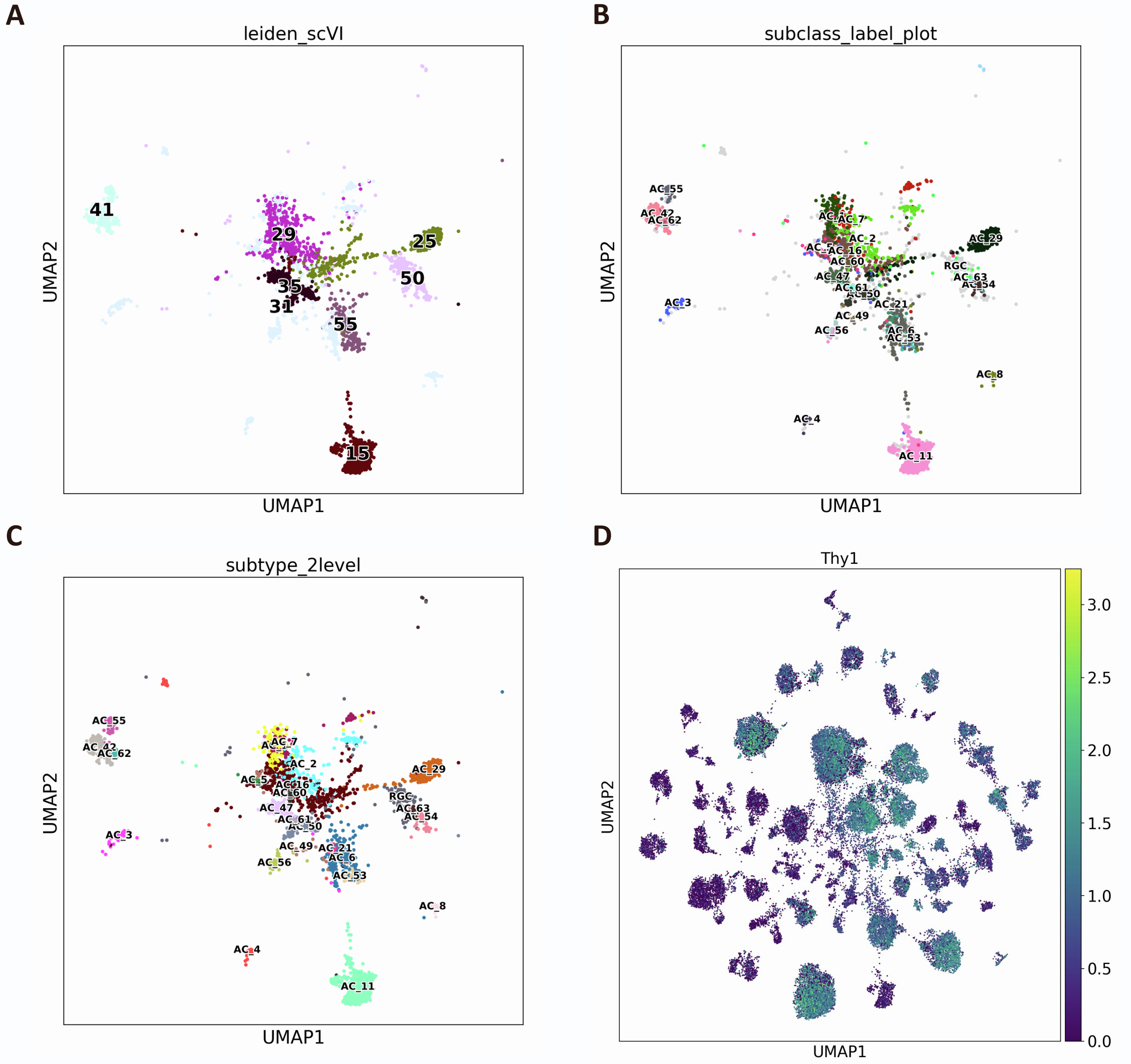

Figure S5. Annotation of over-clustered amacrine cells. Related to Figure 3.
(A) UMAP visualization of the 8 cell clusters in ACs that contain more than one type, based on *Yan et al.* 2020. The 8 clusters are C15, C25, C29, C31, C35, C41, C50, C55. **(B)** UMAP visualization of the 8 clusters colored by public cell type labels from *Yan et al.* 2020. **(C)** UMAP visualization of the 8 clusters colored by AC types using the two-level annotation approach in this study. **(D)** UMAP plot of *Thy1* (CD90) expression in ACs.

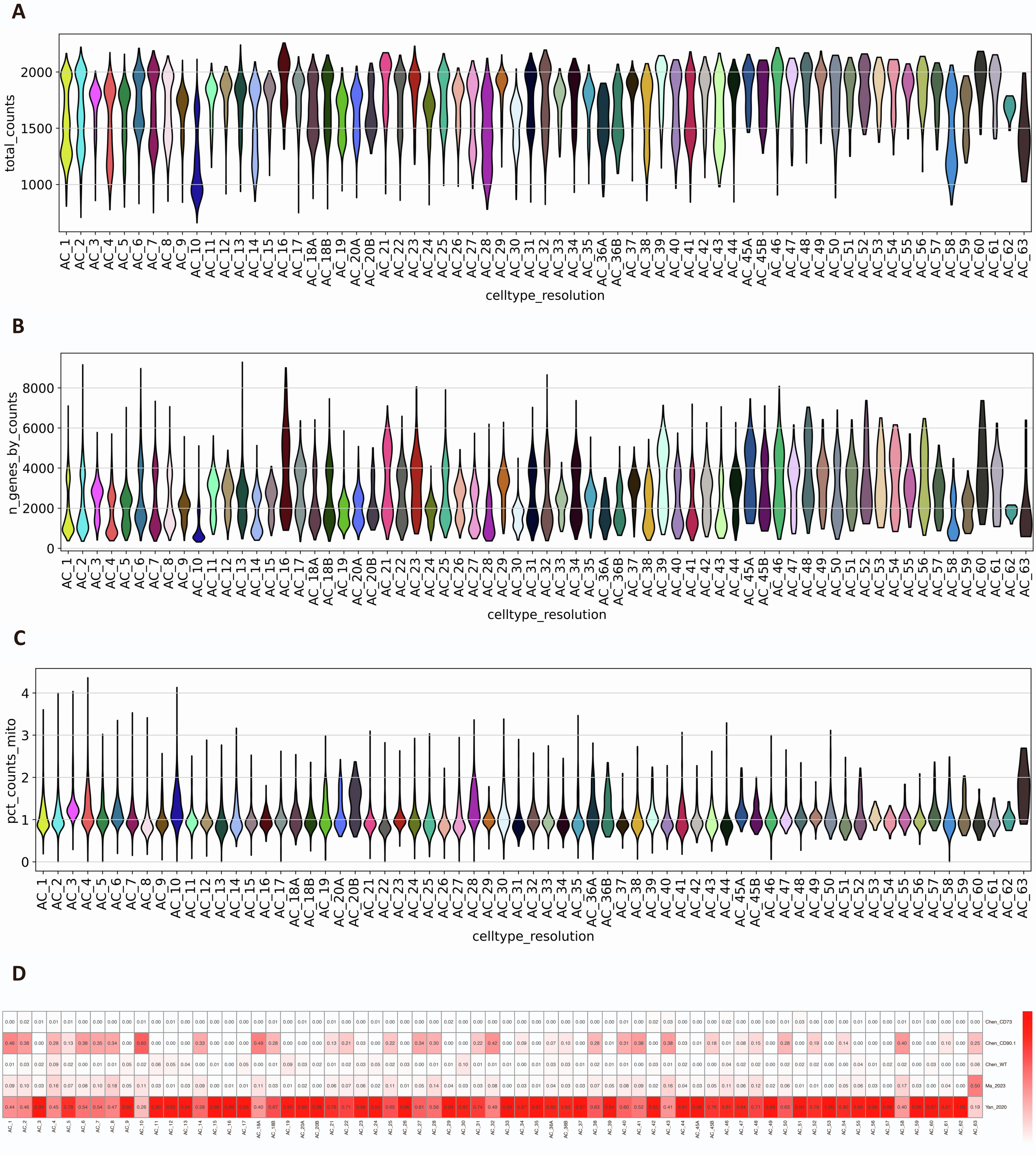

Figure S6. Quality control of AC types. Related to Figure 3 and Figure 4.
Violin plots display the distribution of total UMI counts (**A**), the number of features (**B**), and the percentage of mitochondrial counts (**C**) for the annotated AC types. (**D**) The percentage of dataset sources for AC types. Each row represents a dataset source, while each column represents an AC type. The numbers in the table indicate the percentage of the dataset source for each cell type. The color key denotes the percentages.

A

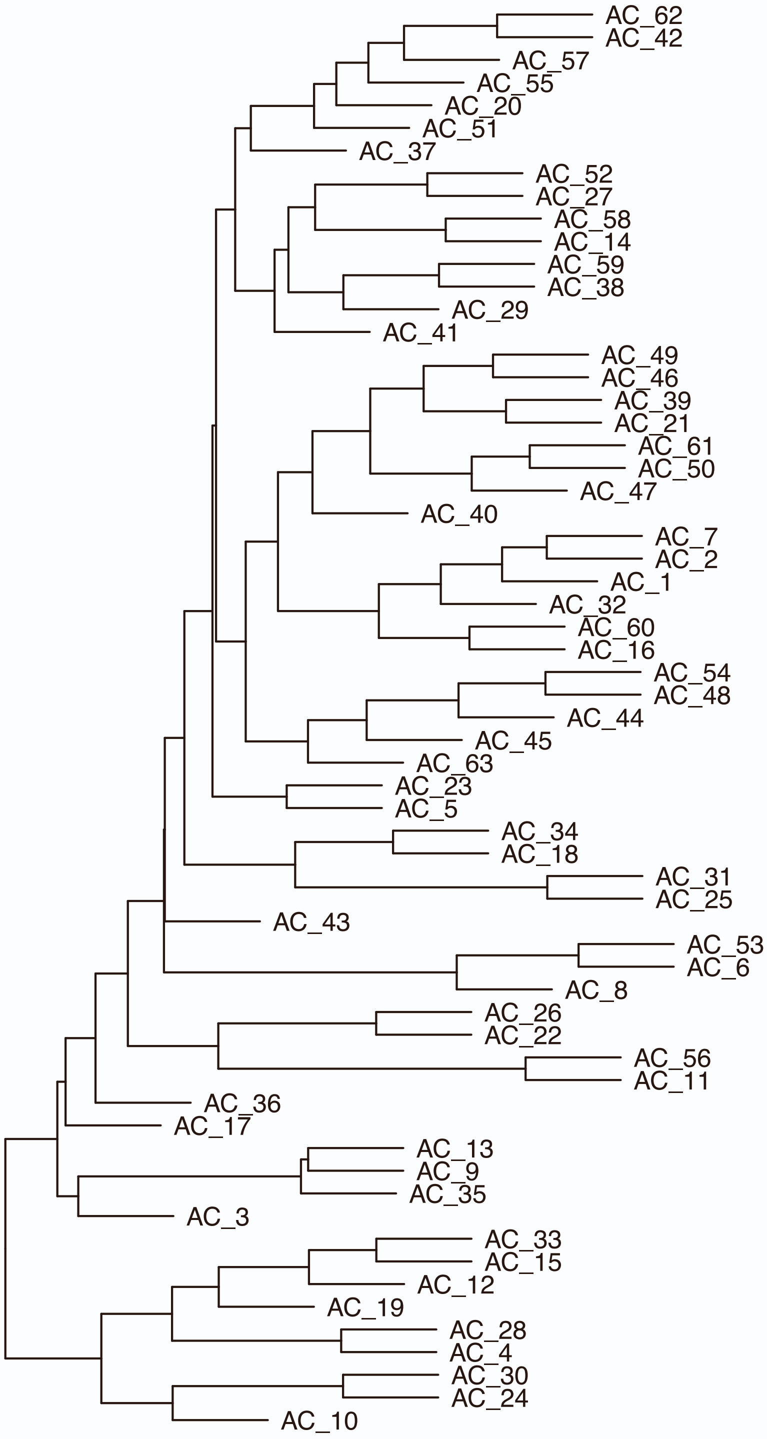

Figure S7. Hierarchical clustering of AC types. Related to Figure 3 and Figure 4.

(**A**) The hierarchical clustering is performed using the average normalized expression for AC types.

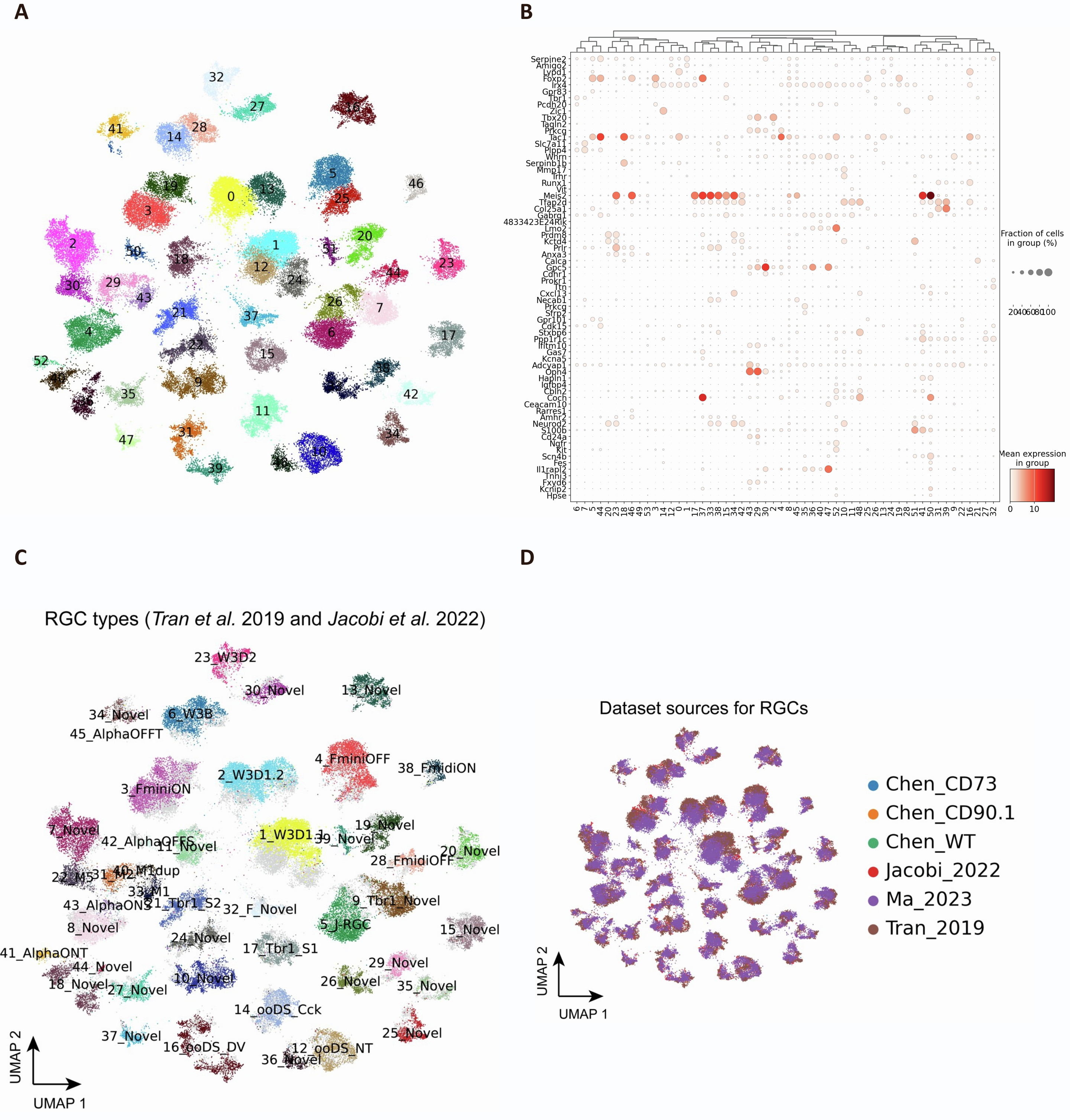

Figure S8. Cell type annotation of retinal ganglion cells. Related to Figure 5.
(A) UMAP visualization of RGCs colored by cluster numbers. **(B)** Dot plot of RGC type marker gene expression in each cluster. **(C)** UMAP visualization of RGCs colored by public cell type labels from *Tran et al.* 2019 and *Jacobi et al.* 2022. The newly discovered cells without public labeling are colored in gray. **(D)** UMAP plot of RGCs colored by data sources.

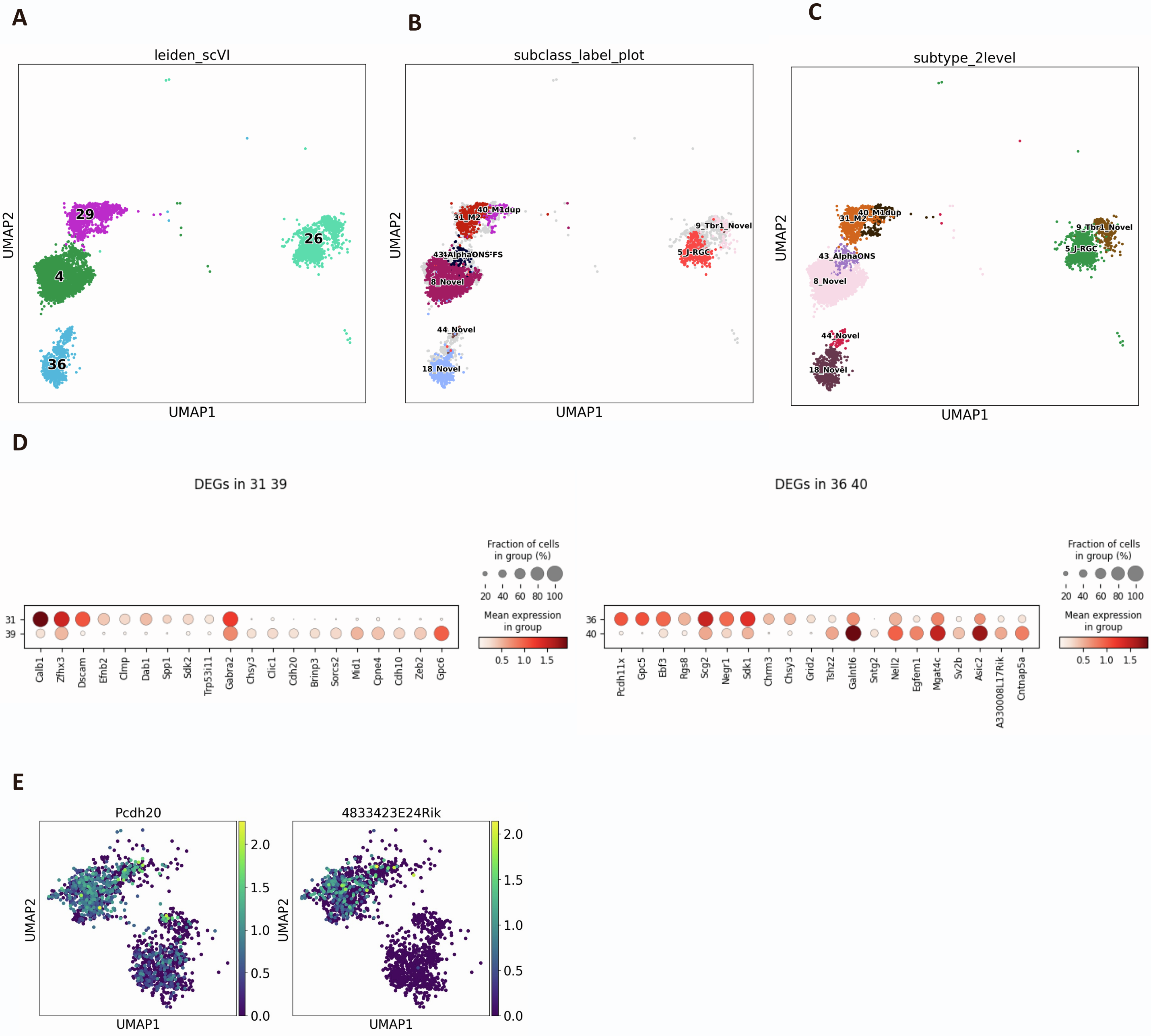

Figure S9. Annotation of over- and under-clustered retinal ganglion cells. Related to Figure 5.
(A) UMAP plot of the 4 clusters in RGCs that contain more than one type, based on *Tran et al.* 2019 and *Jacobi et al.* 2022. **(B)** UMAP plot of the 4 clusters colored by public cell type labels from *Tran et al.* 2019 and *Jacobi et al.* 2022. **(C)** UMAP visualization of the 4 clusters colored by RGC types using the two-level annotation approach in this study. **(D)** Dot plot showing the top 10 differentially expressed genes between split clusters for previously under-clustered RGC types. **(E)** Feature plot showing the marker gene expression of 18_Novel type. Both markers are enriched in one cluster, but not the other.

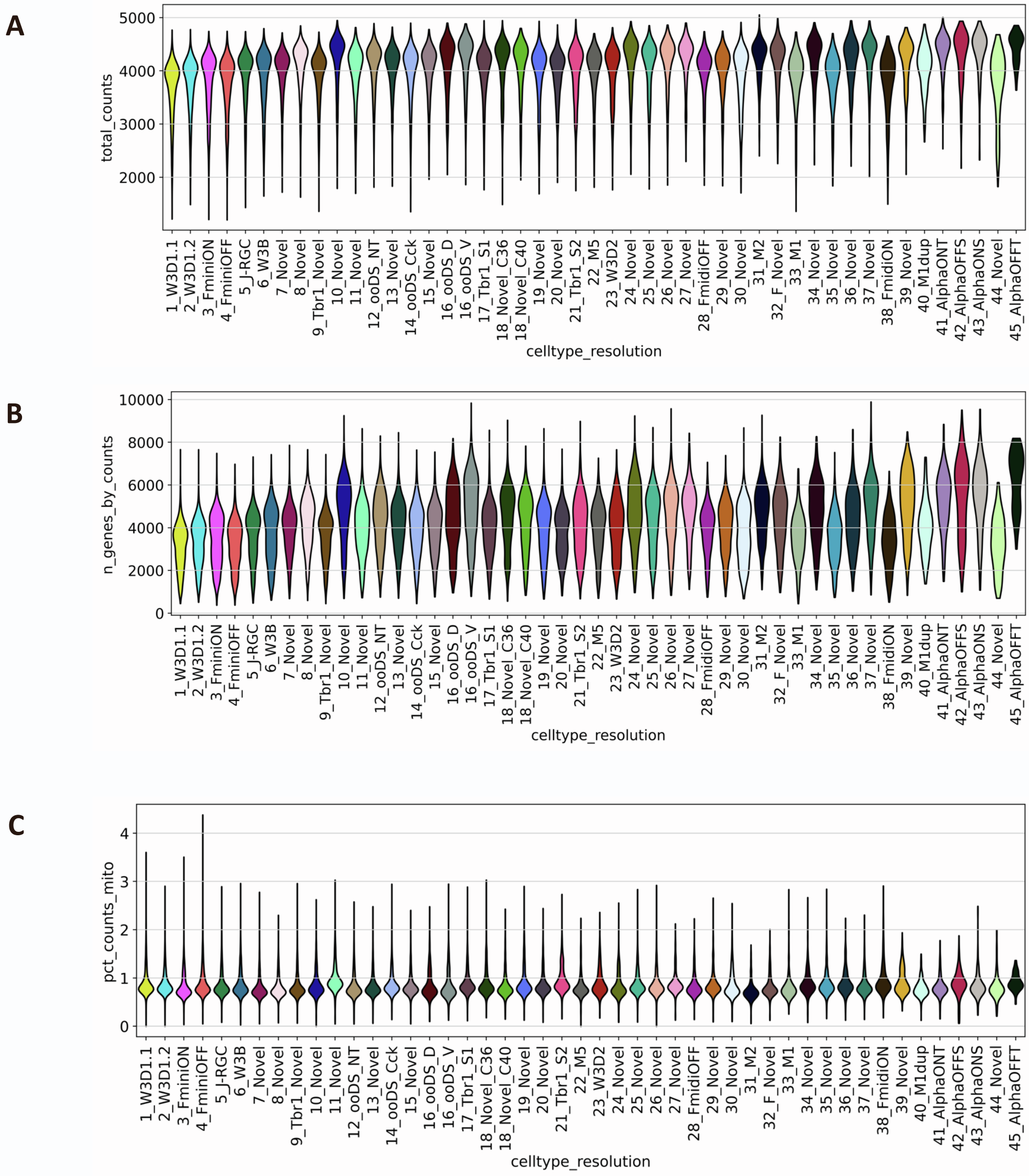

D

	1_W3D1.1	2_W3D1.2	3_FminiON	4_FminiOFF	5_J-RGC	6_W3B	7_Novel	8_Novel	9_Tbr1_Novel	10_Novel	11_Novel	12_ooDS_NT	13_Novel	14_ooDS_Cck	15_Novel
Chen_CD73	0.00	0.00	0.00	0.00	0.00	0.00	0.00	0.00	0.00	0.00	0.00	0.00	0.00	0.00	0.00
Chen_CD90.1	0.02	0.01	0.01	0.01	0.01	0.01	0.00	0.00	0.01	0.00	0.01	0.01	0.01	0.00	0.01
Chen_WT	0.00	0.00	0.00	0.00	0.00	0.00	0.00	0.00	0.00	0.00	0.00	0.00	0.00	0.00	0.00
Jacobi_2022	0.06	0.14	0.16	0.18	0.17	0.17	0.17	0.15	0.14	0.20	0.17	0.19	0.17	0.17	0.19
Ma_2023	0.46	0.36	0.44	0.42	0.37	0.42	0.36	0.42	0.43	0.35	0.40	0.41	0.39	0.48	0.38
Tran_2019	0.45	0.49	0.39	0.40	0.45	0.40	0.46	0.42	0.42	0.44	0.42	0.39	0.43	0.35	0.42

	16_ooDS_D	16_ooDS_V	17_Tbr1_S1	18_Novel_C36	18_Novel_C40	19_Novel	20_Novel	21_Tbr1_S2	22_M5	23_W3D2	24_Novel	25_Novel	26_Novel	27_Novel	28_FmidiOFF
Chen_CD73	0.00	0.00	0.00	0.00	0.00	0.00	0.00	0.00	0.00	0.00	0.00	0.00	0.00	0.00	0.00
Chen_CD90.1	0.00	0.00	0.01	0.00	0.00	0.01	0.02	0.00	0.01	0.00	0.00	0.01	0.01	0.00	0.01
Chen_WT	0.00	0.00	0.00	0.00	0.00	0.00	0.00	0.00	0.00	0.00	0.00	0.00	0.00	0.00	0.00
Jacobi_2022	0.17	0.23	0.18	0.15	0.14	0.19	0.16	0.18	0.15	0.16	0.19	0.17	0.16	0.15	0.10
Ma_2023	0.49	0.45	0.41	0.40	0.47	0.39	0.44	0.44	0.43	0.39	0.50	0.42	0.44	0.44	0.32
Tran_2019	0.33	0.30	0.40	0.45	0.38	0.41	0.38	0.38	0.40	0.44	0.32	0.40	0.38	0.41	0.57

	29_Novel	30_Novel	31_M2	32_F_Novel	33_M1	34_Novel	35_Novel	36_Novel	37_Novel	38_FmidiON	39_Novel	40_M1dup	41_AlphaONT	42_AlphaOFFS	43_AlphaONS	44_Novel	45_AlphaOFFT
Chen_CD73	0.00	0.00	0.00	0.00	0.01	0.00	0.01	0.00	0.01	0.00	0.00	0.00	0.00	0.00	0.00	0.00	0.00
Chen_CD90.1	0.01	0.01	0.01	0.00	0.05	0.00	0.02	0.00	0.00	0.01	0.00	0.06	0.00	0.00	0.00	0.01	0.00
Chen_WT	0.00	0.00	0.00	0.00	0.01	0.00	0.00	0.00	0.00	0.00	0.00	0.00	0.00	0.00	0.00	0.01	0.00
Jacobi_2022	0.13	0.17	0.14	0.22	0.09	0.15	0.09	0.20	0.19	0.17	0.20	0.11	0.23	0.00	0.14	0.09	0.00
Ma_2023	0.40	0.49	0.47	0.42	0.54	0.50	0.51	0.38	0.46	0.56	0.34	0.52	0.32	0.66	0.49	0.61	0.40
Tran_2019	0.45	0.33	0.38	0.35	0.31	0.35	0.37	0.42	0.35	0.26	0.46	0.30	0.44	0.34	0.37	0.28	0.60

Color key: 0.66 – 0

Figure S10. Quality control of RGC types. Related to Figure 5.

Violin plots display the distribution of total UMI counts (**A**), the number of features (**B**), and the percentage of mitochondrial counts (**C**) for the annotated RGC types. (**D**) The percentage of dataset sources for RGC types. Each row represents a dataset source, while each column represents an RGC type. The numbers in the table indicate the percentage of the dataset source for each cell type. The color key denotes the percentages.

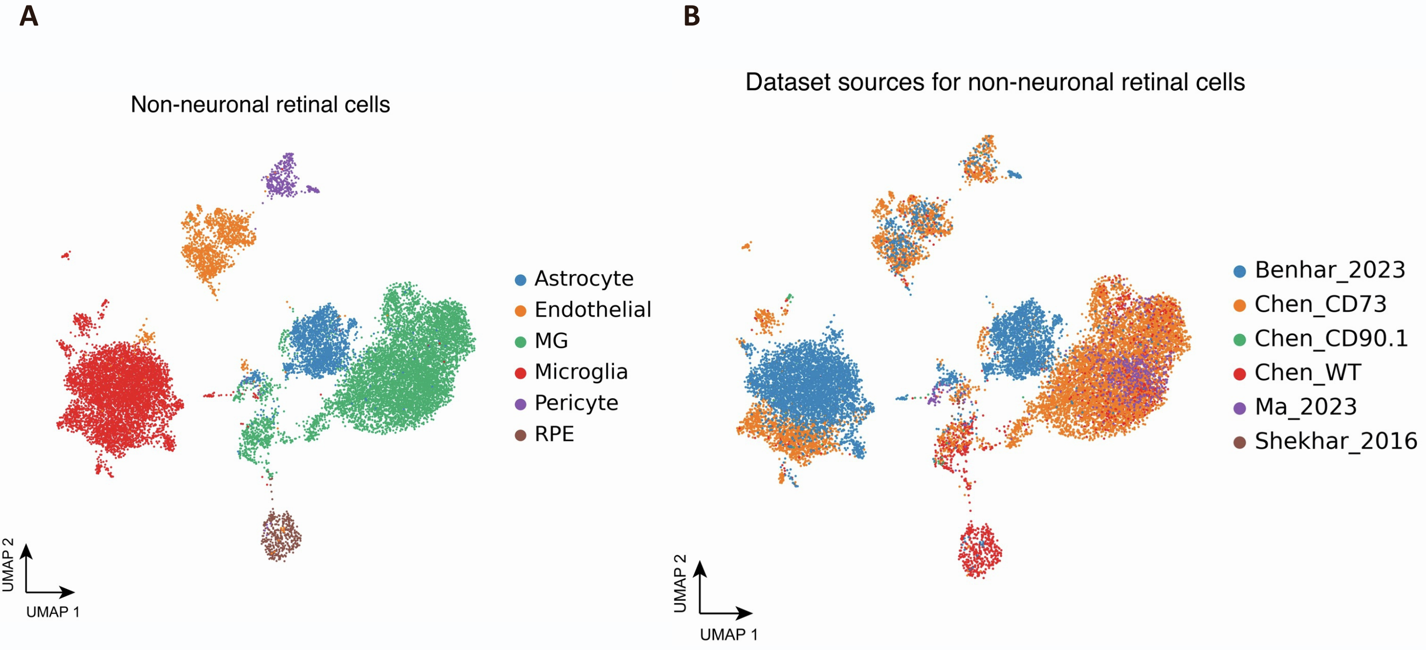

Figure S11. Non-neuronal retinal cells. Related to Figure 6.
(A) UMAP visualization of non-neuronal retinal cells colored by major classes. **(B)** UMAP plot of non-neuronal retinal cells colored by dataset sources.
